# Current situation and trends of radiation therapy in Japan based on the National Database Open Data

**DOI:** 10.1093/jrr/rrae078

**Published:** 2024-10-11

**Authors:** Shohei Okazaki, Masumi Murata, Yoshizumi Kitamoto

**Affiliations:** Department of Radiology, Gunma Prefectural Cancer Center, 617-1, Takahayashinishi, Ota, Gunma, 373-8550, Japan; Department of Radiology, Gunma Prefectural Cancer Center, 617-1, Takahayashinishi, Ota, Gunma, 373-8550, Japan; Department of Radiology, Gunma Prefectural Cancer Center, 617-1, Takahayashinishi, Ota, Gunma, 373-8550, Japan

**Keywords:** national database, open data, radiation therapy, intensity-modulated radiation therapy

## Abstract

This study aimed to visualize the current situation and trends in radiation therapy in Japan using open data from the Japanese National Database of Health Insurance Claims and Specific Health Checkups (NDB). We downloaded the NDB open data from the website of Japan’s Ministry of Health, Labor and Welfare and used Python libraries to analyze the receipt data related to radiation therapy from fiscal year 2014 to 2022. The number of radiation therapy plans peaked in 2019, temporarily declined and subsequently showed a gradual increase. Conversely, the total points associated with radiation therapy have consistently increased without any decline. The use of high-precision radiation therapies such as intensity-modulated radiation therapy (IMRT) has increased over time. Significant regional differences exist, with the Chubu and Kyushu regions showing higher total points and receipts per certified radiation oncologist. A correlation was observed between the number of IMRT plans per population and the number of certified radiation oncologists. Males exhibited a sharp peak in their early 70s, while females demonstrated a mild peak from their 40s to 80s. In recent years, the points for males in their early 70s have rapidly increased. We used the NDB open data to illustrate the current situation and trends in radiation therapy in Japan, highlighting reduced costs and workloads. This study underscored the regional differences in radiation therapy and emphasized the need to discuss strategies for meeting future demand.

## INTRODUCTION

Radiation therapy is a major treatment option for cancer as it can target any organ with minimal invasiveness, making it a vital component of cancer treatment strategies [1, 2]. However, although more than half of patients with cancer in Western countries receive radiation therapy [3, 4], only 20–30% of patients in Japan are treated with this modality [5]. Moreover, Japan faces a significant challenge due to the shortage of radiation oncologists compared with the general population [5]. Understanding the current situation and trends in radiation therapy in Japan is essential to improve accessibility and reduce regional disparities. Every 2 years, the Japanese Society for Radiation Oncology (JASTRO) conducts a nationwide survey of medical facilities to collect data on various aspects of radiation therapy, such as patient numbers, treatment methods and treatment locations [6]. The survey provided valuable insights into the current situation and trends in radiation therapy in Japan. However, the manual data collection process can result in significant human workloads and inconsistencies in aggregation methods among institutions.

As digitalization has become more widespread in recent years, the demand for open data, which are machine-readable information that are freely accessible and usable by anyone, whether for profit or not, is growing. In Japan, national and local governments have made open data available. The Japanese National Database of Health Insurance Claims and Specific Health Checkups (NDB), a large-scale national administrative receipt database launched by Japan’s Ministry of Health, Labor and Welfare (MHLW) in 2009, encompasses data from almost all (99%) hospitals in Japan [7]. The NDB, which has published data on health insurance receipts and specific health checkups since 2011, initially restricted access to researchers. However, in 2016, it began offering the NDB open data, a free-access version of the database available to the public, in response to the increasing demand for open data. Currently, the NDB open data from fiscal year (FY) 2014 onward are available [8].

The NDB open data provide information on receipts for radiation therapy. Receipts can reflect the current status and patterns of radiation therapy in Japan, although the specific treatment details may be difficult to comprehend. This study used open data, especially NDB open data, to present the status and trends of radiation therapy in Japan, highlighting reductions in cost and workload while exploring new insights.

## MATERIALS AND METHODS

### Study design and data gathering

This study was approved by the Institutional Review Board of Gunma Prefectural Cancer Center (registration number: 405-06001). Anonymous open data obtained online were used for the analysis, eliminating the need for informed consent from patients. This study adhered to all relevant laws and guidelines.

Receipt data on radiation therapy from FY 2014 to FY 2022 were obtained from the NDB open data available on the MHLW website. The analysis used data categorized by sex, age and prefecture. An overview of the classification codes for receipts in Japan is provided in Table 1. The codes included M000, for radiation therapy management fee; M001, for external beam radiation therapy; M002, for total body irradiation; M003, for electromagnetic hyperthermia; and M004, for brachytherapy. The M005 code, which indicates blood irradiation, was excluded from the analysis. Each classification code describes the procedures in detail, with points assigned based on each procedure to calculate the receipt.

**Table 1:** Classification codes and their corresponding classification names

Classification codes	Name
M000	Radiation therapy management fee
M000–2	Radioisotope therapy management fee
M001	External beam radiation therapy
M001–2	Stereotactic radiotherapy with Gamma Knife
M001–3	Radiation therapy with linear accelerator
M001–4	Particle beam therapy
M002	Total body irradiation
M003	Electromagnetic hyperthermia
M004	Brachytherapy

Each classification code describes the medical procedures in detail, and points are assigned based on each medical procedure to compute the number of receipts.

To ensure that the data analysis using the NDB accurately reflected reality, the outcomes of the JASTRO structural survey were retrieved from the JASTRO website and used as reference data [6]. The population data by prefecture were obtained from the Statistics Bureau of the Ministry of Internal Affairs and Communications website [9], and the data on certified radiation oncologists (as of 25 December 2023) were sourced from the JASTRO website [10].

### Data visualization and analysis

The data were aggregated, analyzed and visualized using Python (version 3.10.12). The Pandas (version 1.5.3) and Numpy (version 1.25.2) libraries were used for data manipulation, analysis and computation. The Matplotlib library (version 3.7.1) was used for data visualization, while the Japanese map library (version 0.2.0) was used to create a custom map of Japan. The SciPy library (version 1.11.4) was used for statistical analysis. A *P*-value of <0.05 was considered significant.

## RESULTS

### Overall trend

In FY 2022, the latest year with available data, 9 816 129 receipts were recorded, with outpatient services comprising 76.95% of the total. The total number of points for radiation therapy reached 14 708 981 820. Supplementary Fig. 1 and Supplementary Table 1 present the changes in the total number of receipts and the distribution of classification codes over time. Most receipts across all years, except those in FY 2014 (when addition data were missing), were for M001 treatment and the associated additions. The number of M001 treatment remained relatively constant over time, while that of additions gradually increased. Further analysis of the additions showed that they primarily included image-guided procedures, outpatient radiation therapy and particle beam therapy indications (Supplementary Fig. 2).

**Fig. 1:**
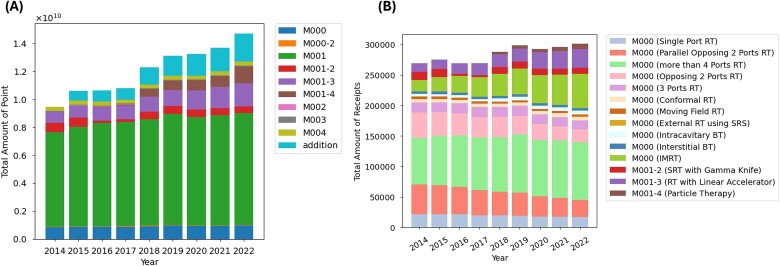
(A) Annual changes in total points for radiation therapy by classification codes. The total points have increased over the years, with a rise in the codes M001, M001–3, M001–4 and additions. (B) Annual changes and distribution of the number of receipts for M000, M001–2, M001–3 and M001–4. The number of receipts temporarily decreased in FY 2020, but eventually increased. Although the use of two-port irradiation has a decreasing trend, the use of irradiation with four or more ports, IMRT, SRT and PBT has shown an increasing trend. Abbreviations: RT = radiation therapy, SRS = sealed radioactive sources, BT = brachytherapy, IMRT = intensity-modulated radiation therapy, SRT = stereotactic radiation therapy.

The annual changes in the total number of points associated with radiation therapy and a breakdown of the classification codes are shown in Fig. 1A and Table 2. The figure indicates a steady increase in the total points over time, with no decrease observed during the coronavirus disease 2019 (COVID-19) pandemic [11]. The breakdown of classification codes revealed an increase in the number of M001, M001–3 (radiation therapy with a linear accelerator, i.e. stereotactic radiation therapy [SRT]), M001–4 (particle beam therapy [PBT]) and additions.

**Table 2:** Annual changes in total points for radiation therapy by classification codes

	FY 2014	FY 2015	FY 2016	FY 2017	FY 2018	FY 2019	FY 2020	FY 2021	FY 2022
M000	840 688 400	867 856 200	888 607 500	892 572 500	920 778 000	964 630 400	939 188 600	953 186 500	970 112 500
M000–2	37 977 730	38 079 770	36 953 480	38 944 940	47 371 210	45 426 720	38 393 390	38 181 010	38 048 600
M001	6 772 470 140	7 116 716 730	7 405 464 780	7 464 479 280	7 595 937 480	7 953 782 640	7 767 258 540	7 870 985 520	8 010 273 660
M001–2	655 850 000	668 550 000	160 250 000	171 200 000	565 450 000	556 175 000	516 150 000	502 600 000	483 050 000
M001–3	815 389 000	848 520 000	937 515 000	1 006 178 000	1 084 035 000	1 156 534 000	1 394 209 000	1 530 943 000	1 660 834 000
M001–4	0	0	48 150 000	54 000 000	510 380 000	668 360 000	707 362 500	753 017 500	1 185 130 000
M002	26 340 000	27 630 000	27 360 000	26 190 000	25 320 000	25 320 000	28 230 000	25 140 000	25 440 000
M003	70 461 000	71 631 000	72 087 000	69 873 000	67 323 000	66 054 000	66 495 000	64 533 000	67 206 000
M004	262 247 480	284 842 000	280 403 360	265 973 000	270 715 360	269 130 960	268 550 640	267 927 240	279 561 480
Additions	0	690 228 960	784 167 540	829 435 640	1 222 935 340	1 437 725 820	1 557 082 310	1 683 342 180	1 989 325 580

These data correspond to the graph in Fig. 1A.

Figure 1B and Table 3 present the annual changes and breakdowns in the number of receipts for M000, M001–2 (stereotactic radiotherapy with Gamma Knife), M001–3 and M001–4. M000 is billed once for each radiation therapy plan when M001 and M004 treatments are performed. Therefore, the number of M000 receipts roughly corresponds to the number of radiation therapy plans for M001 and M004 treatments. M001–2, M001–3 and M001–4 treatments are not billed with M000, but are billed once for each course of treatment. Therefore, the number of receipts is expected to correspond to the number of treatment plans, except when multiple treatment plans are made in a course of treatment. The total number of these receipts decreased temporarily in FY 2020, which coincided with the emergence of the COVID-19 pandemic, but subsequently increased. The breakdown revealed that irradiation with two ports was decreasing, while irradiation with more than four ports, intensity-modulated radiation therapy (IMRT), SRT and PBT increased.

**Table 3:** Annual changes and distribution in the number of receipts for M000, M001–2, M001–3 and M001–4

		FY 2014	FY 2015	FY 2016	FY 2017	FY 2018	FY 2019	FY 2020	FY 2021	FY 2022
M000	Single-port RT	21 913	21 752	21 533	19 960	19 695	19 262	17 929	17 474	16 840
	Parallel opposing 2-ports RT	48 342	47 550	44 918	41 272	38 930	37 912	33 494	30 728	27 995
	More than 4-port RT	76 376	80 730	84 551	86 190	89 546	95 060	91 890	94 570	94 738
	Opposing 2-port RT	41 355	38 692	35 830	33 681	33 112	30 470	26 033	22 959	21 345
	Three-port RT	17 134	16 529	16 890	16 781	16 447	16 672	15 912	15 465	14 950
	Conformal RT	5381	5067	5497	5386	5514	5611	5573	6241	6027
	Moving field RT	4129	3734	3373	3327	3088	3384	3261	3189	3283
	External RT using SRS	718	768	337	291	264	252	233	219	96
	Intracavitary BT	3686	3719	3887	3568	5156	5692	5585	5918	6083
	Interstitial BT	3900	4254	4096	3926	4228	4282	3911	3888	4195
	IMRT	18 876	23 454	27 819	32 123	36 066	41 894	46 170	50 066	56 359
M001–2	SRT with gamma knife	13 117	13 371	3205	3424	11 309	11 063	10 323	10 052	9661
M001–3	RT with linear accelerator	14 453	15 315	17 165	18 701	20 245	21 958	26 718	28 781	31 288
M001–4	Particle therapy	0	0	321	360	4017	5363	5645	6084	8742

These data correspond to the graph in Fig. 1B.

Abbreviations: RT = radiation therapy, SRS = sealed radioactive sources, BT = brachytherapy, IMRT = intensity-modulated radiation therapy, SRT = stereotactic radiation therapy

**Fig. 2:**
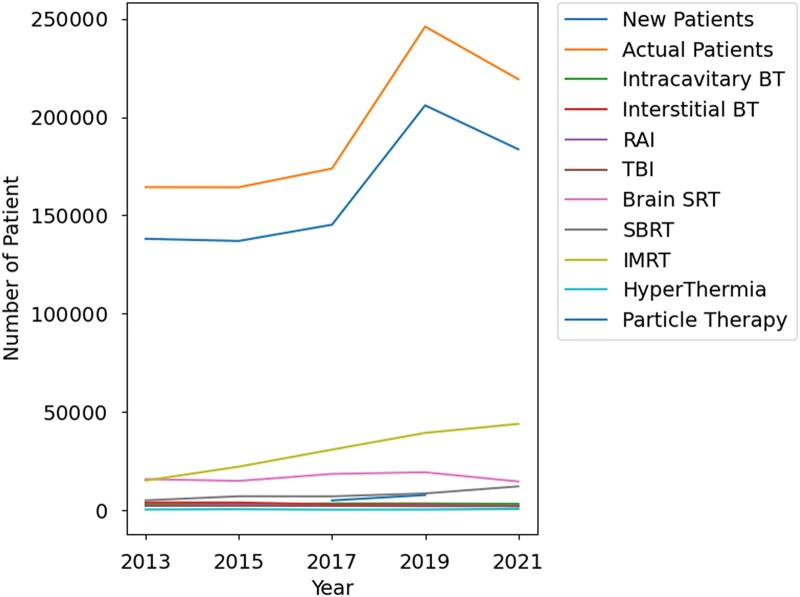
The line graph illustrates the annual changes in the number of treatments based on the JASTRO structural survey. The number of radiation therapy patients has been declining since 2019, when it reached its highest. IMRT shows a rising trend. Only the 2017 and 2019 surveys reported the number of particle beam therapy. Abbreviations: BT = brachytherapy, RAI = radioactive iodine therapy, TBI = total body irradiation, SRT = stereotactic radiation therapy, SBRT = stereotactic body radiation therapy, IMRT = intensity-modulated radiation therapy.

The line graph in Fig. 2 and Supplementary Table 2 illustrate the changes in the number of treatments based on the JASTRO structural survey. Although the count was made biennially, the number of patients receiving radiation therapy has decreased since its peak in 2019. An increase in the number of IMRT sessions was also observed. These trends align with the changes in the number of specific receipts recorded in the NDB open data.

### Disparity across prefectures

**Fig. 3:**
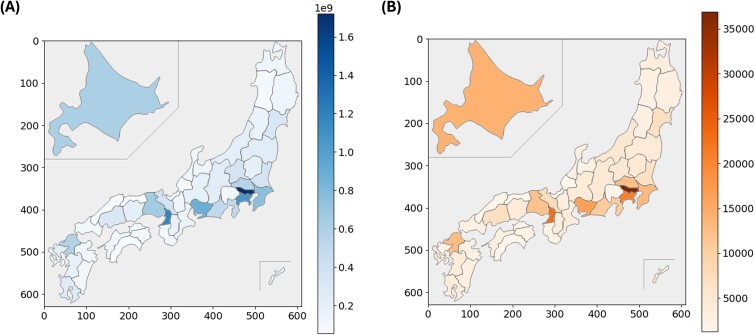
(A) Distribution of total points for radiation therapy in different prefectures. (B) Distribution of the number of receipts for M000, M001–2, M001–3 and M001–4 in different prefectures. Both panels show higher numbers in the urban prefectures, including Tokyo.

**Fig. 4:**
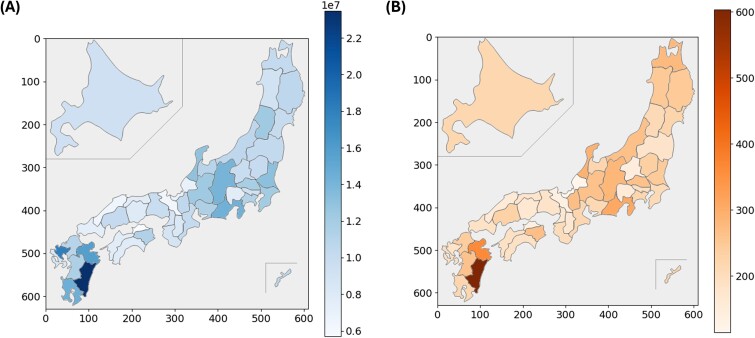
(A) Distribution of total points for radiation therapy for every enrolled certificated radiation oncologist in each prefecture. (B) Distribution of the number of receipts for M000, M001–2, M001–3 and M001–4 for every enrolled certificated radiation oncologist in each prefecture. Both panels show higher numbers in the Chubu and Kyushu regions, while the numbers were lower in Tokyo.

Figure 3A illustrates the distribution of the total number of points related to radiation therapy across the prefectures. Figure 3B displays the number of receipts for M000, M001–2, M001–3 and M001–4 across the prefectures. Both panels indicate a higher number in the prefectures with urban areas, including Tokyo. Figure 4A and B presents the results normalized by the number of certified radiation oncologists enrolled. The Chubu and Kyushu regions had higher numbers, while Tokyo had lower numbers.

**Fig. 5:**
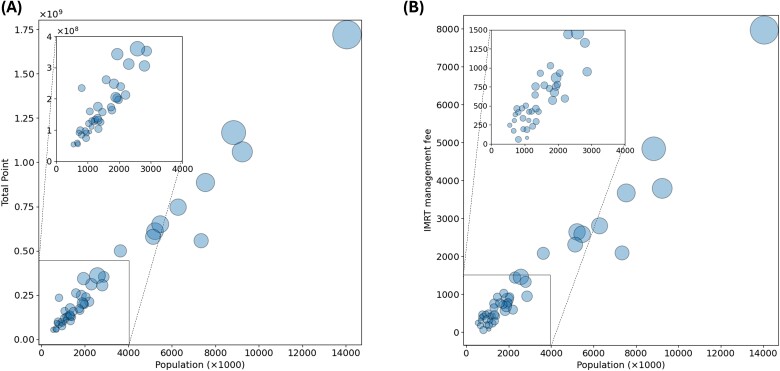
(A) A scatter plot showing the relationship between population and radiation therapy points in each prefecture. The plot size reflects the number of certificated radiation oncologists in a specific prefecture. (B) Scatter plot showing the relationship between population and IMRT management fee receipts in each prefecture. The number of IMRT plans was lower in prefectures with fewer certificated radiation oncologists than in prefectures with similar population sizes.

Figure 5A depicts the relationship between the population of each prefecture and the total number of points assigned for radiation therapy. The size of the plot shows the number of certified radiation oncologists in each prefecture. The number of radiation oncologists in each prefecture did not influence the positive linear relationship between population size and total points. Figure 5B presents a scatter plot illustrating the relationship between population size and the number of IMRT receipts, which represents high-precision treatments. Compared with prefectures with similar population sizes, those with fewer certified radiation oncologists exhibited a slight decrease in the rate of IMRT plans. Spearman’s rank correlation test showed a weak but significant correlation between the number of IMRT plans per population and the number of certified radiation oncologists, with a *P*-value of 0.01 and a correlation coefficient of 0.369.

### Distribution by patients’ sex and age

**Fig. 6:**
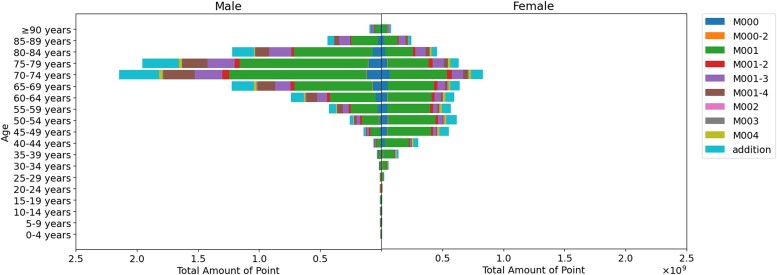
The points for radiation therapy are distributed differently by patients’ sex and age. Males showed a significant peak in their 70s, while females showed a mild peak from 40 to 80 years old. In both sexes, M001 accounts for the majority of points in every age group.

Figure 6 and Supplementary Table 3 display the total points according to patients’ sex and age, based on the FY 2022 data. Males showed a sharp peak in their 70s, while females showed a mild peak between the ages of 40 and 80 years. The M001 category predominated across all sexes and age groups. Supplementary Fig. 3 provides a graphical animation depicting the changes in the total points by patients’ sex and age over time, indicating a rapid increase, especially among males in their early 70s.

## DISCUSSION

The NDB open data are a resource of health data that have been anonymized and made available on the website, but it offers a lot of potential for analysis depending on the researcher’s concept. These data can be combined with other sources, such as open data and literature meta-analyses, to facilitate more comprehensive and in-depth research [12, 13]. The NDB open data have been used in several research projects on radiation therapy. Katano *et al*. analyzed the NDB open data from 2015 to 2019 and reported an increase in the number of receipts involving radiation therapy and the number of IMRT based on the M000 receipts in that period [14]. Using open NDB and COVID-19 data, Tamari *et al*. showed that areas experiencing a COVID-19 outbreak had a lower rate of conventionally fractionated radiation regimens and a higher rate of hypofractionated radiation regimens [15]. Although some of the content in this study overlaps with those of previous research, our analyses were conducted from different perspectives, and we believe that the results of this study are valuable.

We visualized the NDB open data from three perspectives: overall trends, disparity across prefectures and distribution by patients’ sex and age. The overall trend indicated that the number of radiation therapy plans, based on the M000, M001–2, M001–3 and M001–4 receipts, peaked in FY 2019, temporarily declined and subsequently increased. Conversely, the total points for radiation therapy exhibited a steady increase, with no observed decrease. The COVID-19 pandemic may have initially reduced the number of radiation therapy plans; however, the increasing use of high-precision radiation therapies, such as IMRT, SRT and PBT, likely contributed to the higher total points. This idea is supported by the increasing points of additions, particularly those involving image-guided plans. This trend aligns with the findings of the JASTRO structural survey, suggesting that the NDB open data analysis could complement certain aspects of structural surveys. Given that the NDB open data are updated annually, they offer the potential for more detailed analysis compared with the biennial nature of structural surveys. Despite the increase in total points associated with radiation therapy, the most recent FY recorded approximately 14.7 billion points. Assuming one point corresponds to 10 Japanese yen (JPY), the estimated healthcare costs associated with radiation therapy amount to 147 billion JPY. The national healthcare expenditure of Japan in FY 2021 was approximately 45.0 trillion JPY, with medical care expenditure for neoplasms (tumors) accounting for 4.8 trillion JPY [16]. Consequently, the expenditure on radiation therapy represents a small proportion of the total. Given its significant role in cancer treatment, radiation therapy may be considered a cost-effective treatment.

When examining the disparity across prefectures, urban areas had a higher number of radiation therapy plans and overall points per prefecture, while rural areas had a higher number of radiation therapy plans per certified radiation oncologist. In rural areas, radiation oncologists may handle multiple treatments due to a shortage of staff, not only among doctors but also among radiation therapy personnel, compared with urban areas [5]. Limited evidence suggests that staff workload directly affects the likelihood of radiation therapy accidents; however, human error remains the primary factor behind these incidents [17]. Without intervention, rural areas could experience a higher incidence of medical accidents, highlighting the need to address regional inequalities. Moreover, prefectures with fewer certified radiation oncologists had a lower number of IMRT plans per capita. IMRT and other high-precision radiation therapies are more time-consuming and labor-intensive than conventional external beam radiation therapy [18]. Consequently, prefectures with few certified radiation oncologists may not offer these advanced high-precision radiation therapies, potentially depriving patients of high-precision radiation treatment. This situation underscores the importance of addressing regional imbalances.

The analysis of distribution by patients’ sex and age showed that males had a sharp peak in total points in their early 70s, while females had a mild peak from their 40s to 80s. This pattern aligns with the higher prevalence of cancer in older men, while women tend to develop cancer at an earlier age [19]. Both sexes showed a decline in total points from their late 70s onward, possibly indicating that older patients may be less likely to seek treatment. Temporal analysis showed a significant increase in the total points for males in their early 70s over the last few years. Prostate cancer is common in older men and often treated with radiation therapy. Patients with prostate cancer typically receive specific radiation therapies such as IMRT, SRT, PBT and brachytherapy [20], which result in higher total points. Therefore, the sharp increase in the total points for men in the early 70s indicates a rise in the number of prostate cancer cases. The demand for radiation therapy for prostate cancer is expected to grow in the future. The application of technologies such as artificial intelligence-based radiation therapy planning [21] may help improve the efficiency of radiation therapy.

The limitation of NDB open data is that they only provide receipt data and specific health checkup data, which do not include patient-specific information (diagnosis, comorbidities, general status, etc.), treatment information (irradiation site, irradiation dose, etc.) and information on medical institutions (facility size, number of staff, etc.). The number of receipts for M000 only provides an approximate number of treatment plans for M001 and M004, while the number of receipts for M001–2, M001–3 and M001–4 only provides an approximate number of treatment course. Considering that some patients require multiple treatment plans for a single course, and that some may receive more than one course of treatment in the same FY, accurately determining the actual number of patients remains challenging. Therefore, the NDB open data cannot completely substitute the information provided by structural surveys. However, as demonstrated in this study, the NDB open data are useful for assessing the current situation and trends of radiation therapy in Japan, potentially offering insights beyond the scope of structural surveys. Moreover, the NDB open data adhere to the principle of minimum aggregation units and exclude datasets with fewer than 10 receipts. Therefore, this study may not have included some receipts in the analysis. However, this is a very small number in the overall picture and does not influence most of the analytical outcomes. Moreover, as the open data do not contain personal information, they are less likely to expose any private data.

We analyzed the status and trends of radiation therapy in Japan using the NDB open data, with a focus on reducing costs and workloads. Despite the temporary decrease in the number of radiation therapy plans, the total number of points for radiation therapy has increased, likely due to the expansion of high-precision radiation therapy. The future demand for radiation therapy, especially for prostate cancer, is expected to increase. However, the uneven distribution of radiation oncologists could lead to potentially heavy workloads in some regions. To expand and advance radiation therapy in Japan, reducing regional imbalances and enhancing operational effectiveness are essential.

## ABBREVIATIONS

NDB, National Database; FY, Fiscal Year; JASTRO, Japanese Society for Radiation Oncology; MHLW, Ministry of Health, Labor and Welfare; IMRT, intensity-modulated radiation therapy; SRT, stereotactic radiation therapy; PBT, particle beam therapy.

## Supplementary Material

FigS1_rrae078

FigS2_rrae078

FigS3_rrae078

Supplementary_Table1_rrae078

Supplementary_Table2_rrae078

Supplementary_Table3_rrae078

## PRESENTATION AT A CONFERENCE

The study is expected to be presented at the 37th annual meeting of the Japanese Society for Radiation Oncology.
