# Supplementary figures and images for: Current situation and trends of radiation therapy in Japan based on the National Database Open Data
Source: J Radiat Res. 2024 Oct 11;65(6):864–71. doi: 10.1093/jrr/rrae078 (PMC11630034; doi:10.1093/jrr/rrae078)

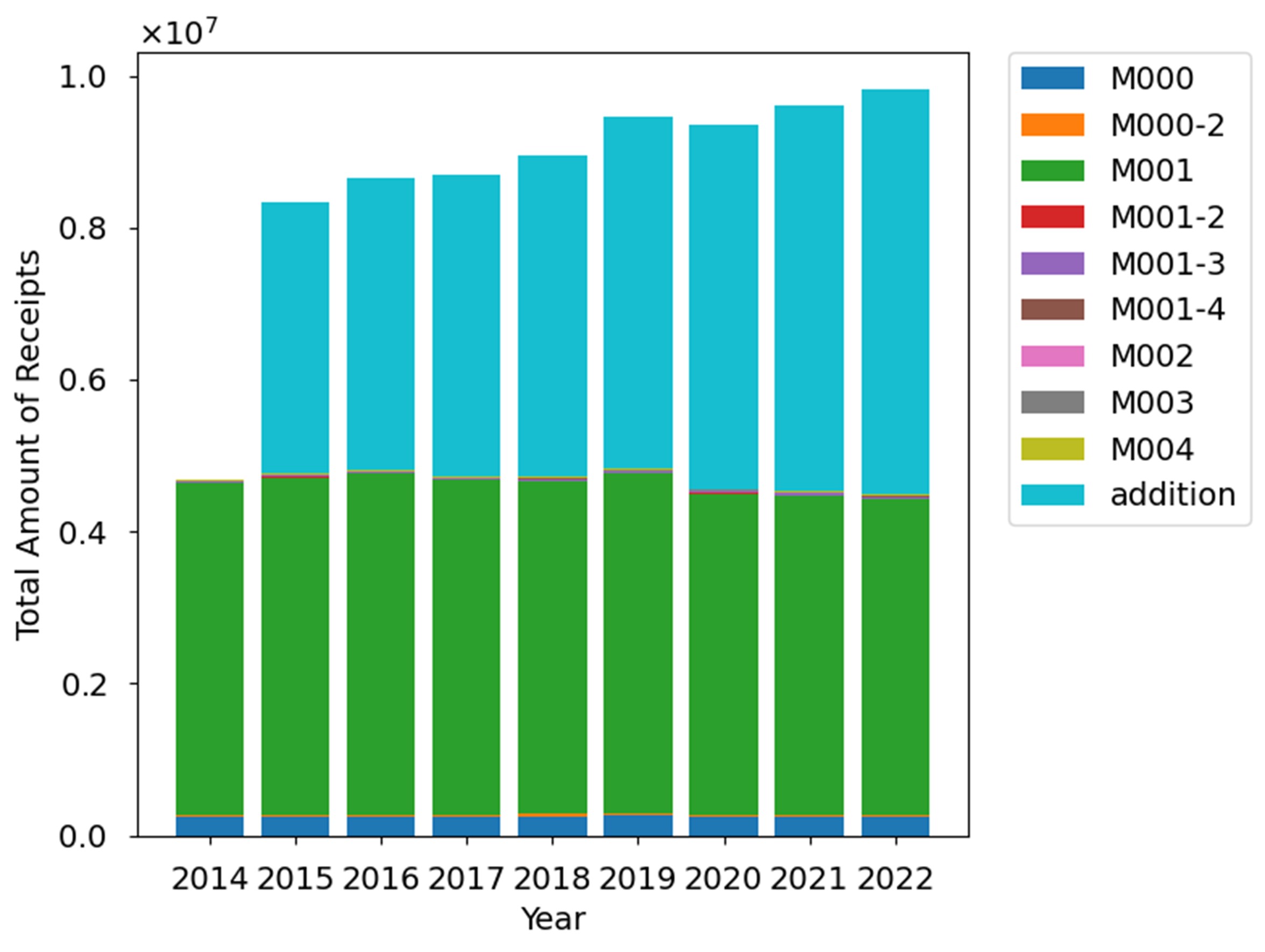

Supplement: FigS1_rrae078 [file figs1_rrae078.jpeg]

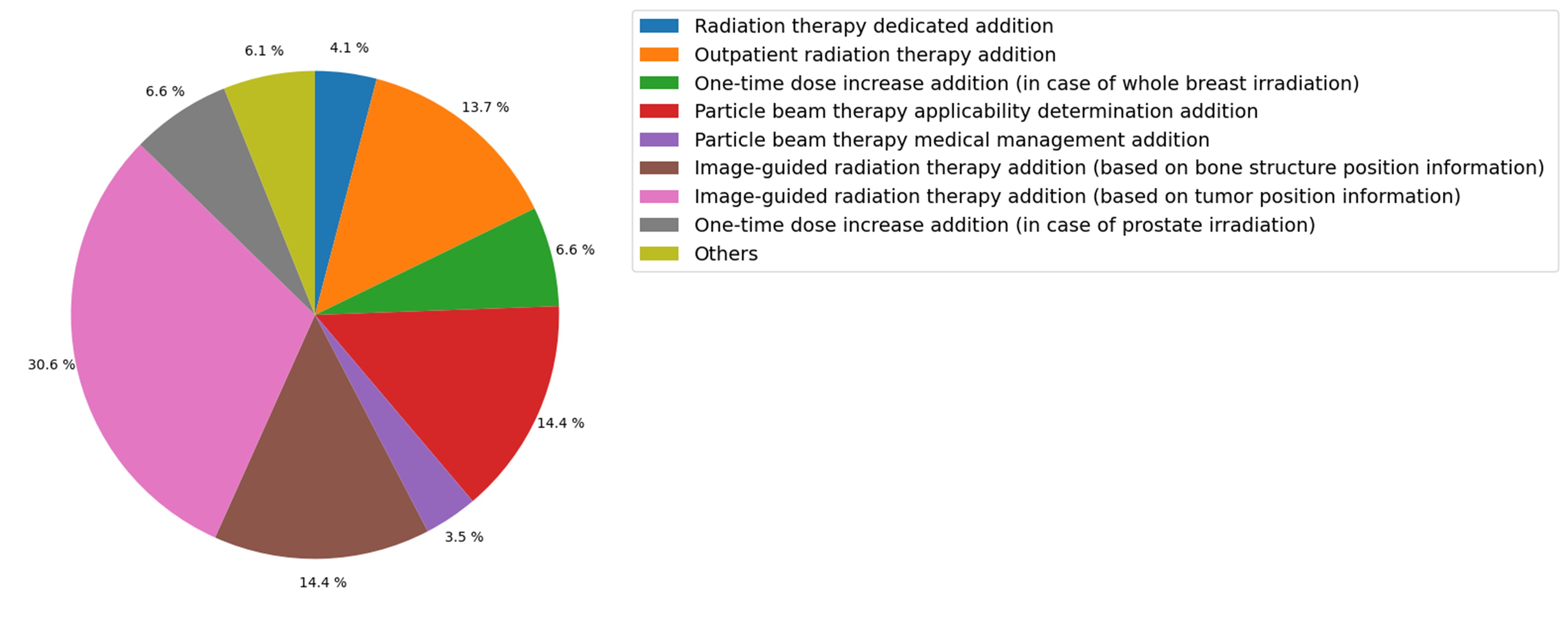

Supplement: FigS2_rrae078 [file figs2_rrae078.jpeg]

## Slide 1
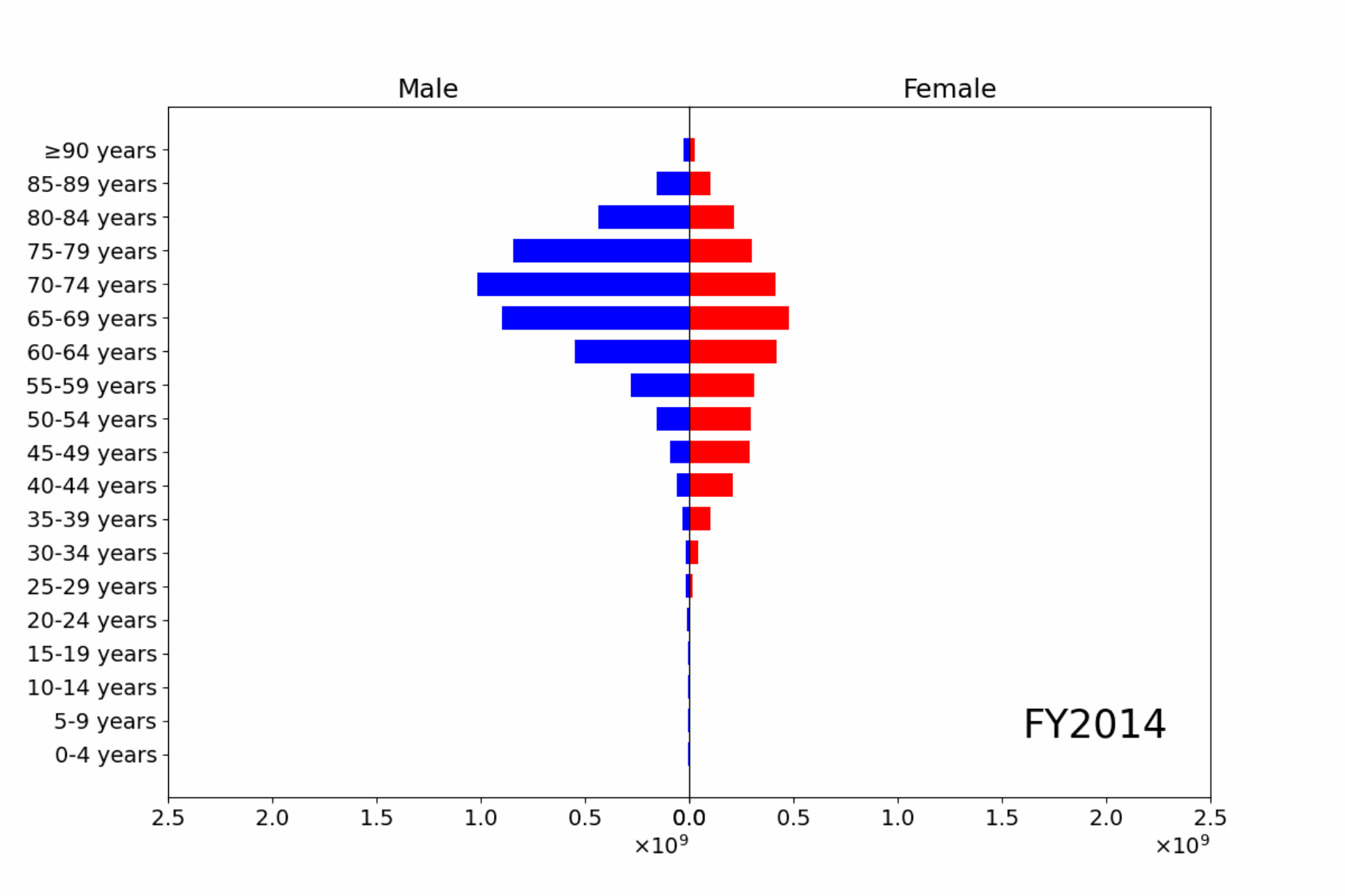

Supplement: FigS3_rrae078 [file figs3_rrae078.pptx]
