# Supplementary material for: Current situation and trends of radiation therapy in Japan based on the National Database Open Data
Source: J Radiat Res. 2024 Oct 11;65(6):864–71. doi: 10.1093/jrr/rrae078 (PMC11630034; doi:10.1093/jrr/rrae078)
Supplement: Supplementary_Table1_rrae078 [file supplementary_table1_rrae078.docx]

**Supplementary Table 1**. Annual changes in the total number of receipts and the distribution of classification codes

|  | FY 2014 | FY 2015 | FY 2016 | FY 2017 | FY 2018 | FY 2019 | FY 2020 | FY 2021 | FY 2022 |
| --- | --- | --- | --- | --- | --- | --- | --- | --- | --- |
| M000 | 241,810 | 246,249 | 248,731 | 246,505 | 252,046 | 260,491 | 249,991 | 250,717 | 251,911 |
| M000–2 | 26,307 | 26,535 | 25,636 | 26,657 | 28,851 | 28,024 | 23,695 | 23,774 | 22,954 |
| M001 | 4,364,697 | 4,441,815 | 4,489,138 | 4,401,045 | 4,380,457 | 4,472,169 | 4,227,673 | 4,190,550 | 4,144,563 |
| M001–2 | 13,117 | 13,371 | 3,205 | 3,424 | 11,309 | 11,063 | 10,323 | 10,052 | 9,661 |
| M001–3 | 14,453 | 15,315 | 17,165 | 18,701 | 20,245 | 21,958 | 26,718 | 28,781 | 31,288 |
| M001–4 | 0 | 0 | 321 | 360 | 4,017 | 5,363 | 5,645 | 6,084 | 8,742 |
| M002 | 878 | 921 | 912 | 873 | 844 | 844 | 941 | 838 | 848 |
| M003 | 8,005 | 8,143 | 8,199 | 7,969 | 7,705 | 7,553 | 7,596 | 7,390 | 7,677 |
| M004 | 13,876 | 14,390 | 14,462 | 13,957 | 14,309 | 14,558 | 13,800 | 14,080 | 14,627 |
| Addition | 0 | 3,558,194 | 3,855,361 | 3,975,849 | 4,220,698 | 4,636,810 | 4,789,638 | 5,072,407 | 5,323,776 |

These data correspond to the graph in Supplementary Figure 1.
