# Supplementary material for: Current situation and trends of radiation therapy in Japan based on the National Database Open Data
Source: J Radiat Res. 2024 Oct 11;65(6):864–71. doi: 10.1093/jrr/rrae078 (PMC11630034; doi:10.1093/jrr/rrae078)
Supplement: Supplementary_Table2_rrae078 [file supplementary_table2_rrae078.docx]

**Supplementary Table 2**. Annual changes in the number of treatments based on the JASTRO structural survey.

|  | 2013 | 2015 | 2017 | 2019 | 2021 |
| --- | --- | --- | --- | --- | --- |
| New patients | 138,076 | 136,987 | 145,243 | 205,964 | 183,613 |
| Actual patients | 164,346 | 164,297 | 173,818 | 246,046 | 219,260 |
| Intracavitary BT | 3,128 | 3,117 | 3,431 | 3,438 | 3,233 |
| Interstitial BT | 3,958 | 3,880 | 2,948 | 2,911 | 2,059 |
| RAI | 2,332 | 2,763 | 2,246 | 2,454 | 2,361 |
| TBI | 2,327 | 2,287 | 2,253 | 2,085 | 2,056 |
| Brain SRT | 15,828 | 14,910 | 18,484 | 19,315 | 14,617 |
| SBRT | 5,023 | 7,104 | 7,072 | 8,554 | 12,145 |
| IMRT | 15,119 | 22,168 | 30,835 | 39,341 | 43,922 |
| Hyperthermia | 366 | 510 | 276 | 327 | 664 |
| Particle therapy | N/A | N/A | 4,997 | 7,728 | N/A |

These data correspond to the graph in Figure 2.

Abbreviations: BT, brachytherapy; RAI, radioactive iodine therapy; TBI, total body irradiation; SRT, stereotactic radiation therapy; SBRT, stereotactic body radiation therapy; IMRT, intensity–modulated radiation therapy; N/A, not available
