# Supplementary material for: Current situation and trends of radiation therapy in Japan based on the National Database Open Data
Source: J Radiat Res. 2024 Oct 11;65(6):864–71. doi: 10.1093/jrr/rrae078 (PMC11630034; doi:10.1093/jrr/rrae078)
Supplement: Supplementary_Table3_rrae078 [file supplementary_table3_rrae078.docx]

**Supplementary Table 3**. Points for radiation therapy by sex and age in FY 2022. (A) Male. (B) Female.

(A)

|  | 0–4 years | 5–9 years | 10–14 years | 15–19 years | 20–24 years | 25–29 years | 30–34 years | 35–39 years | 40–44 years | 45–49 years |
| --- | --- | --- | --- | --- | --- | --- | --- | --- | --- | --- |
| M000 | 205,800 | 336,300 | 386,800 | 656,500 | 968,100 | 887,000 | 1,379,800 | 2,343,600 | 3,983,600 | 9,330,100 |
| M000–2 | 0 | 0 | 0 | 31,970 | 179,310 | 305,800 | 347,690 | 558,010 | 703,840 | 856,830 |
| M001 | 2,065,080 | 3,422,520 | 3,473,400 | 5,369,040 | 6,945,900 | 6,553,080 | 10,879,860 | 19,409,460 | 33,539,520 | 78,967,260 |
| M001–2 | 0 | 750,000 | 650,000 | 1,200,000 | 1,050,000 | 1,800,000 | 1,700,000 | 3,050,000 | 5,550,000 | 9,150,000 |
| M001–3 | 0 | 0 | 0 | 819,000 | 750,000 | 1,372,000 | 2,142,000 | 4,735,000 | 8,106,000 | 17,224,000 |
| M001–4 | 4,125,000 | 3,562,500 | 3,750,000 | 0 | 0 | 0 | 0 | 0 | 5,062,500 | 9,562,500 |
| M002 | 450,000 | 540,000 | 540,000 | 420,000 | 390,000 | 600,000 | 510,000 | 780,000 | 630,000 | 1,590,000 |
| M003 | 0 | 0 | 0 | 0 | 0 | 0 | 126,000 | 279,000 | 909,000 | 1,539,000 |
| M004 | 0 | 0 | 0 | 0 | 0 | 0 | 0 | 0 | 0 | 0 |
| addition | 1,345,450 | 1,347,240 | 1,479,300 | 1,966,160 | 1,021,500 | 986,860 | 1,784,570 | 3,445,280 | 7,105,370 | 15,478,840 |

|  | 50–54 years | 55–59 years | 60–64 years | 65–69 years | 70–74 years | 75–79 years | 80–84 years | 85–89 years | ≥90 years |
| --- | --- | --- | --- | --- | --- | --- | --- | --- | --- |
| M000 | 16,847,900 | 25,838,000 | 42,436,900 | 68,831,000 | 116,976,400 | 104,866,800 | 67,003,900 | 26,696,400 | 7,227,500 |
| M000–2 | 911,060 | 1,017,710 | 1,299,190 | 2,108,010 | 3,772,750 | 3,036,410 | 2,202,870 | 815,570 | 173,580 |
| M001 | 138,996,600 | 222,465,840 | 374,734,200 | 638,410,140 | 1,124,587,140 | 1,051,783,320 | 643,921,860 | 216,254,280 | 47,892,180 |
| M001–2 | 17,150,000 | 17,450,000 | 24,800,000 | 33,200,000 | 53,750,000 | 40,100,000 | 22,900,000 | 11,100,000 | 2,700,000 |
| M001–3 | 28,792,000 | 45,456,000 | 79,339,000 | 125,831,000 | 229,271,000 | 221,365,000 | 182,190,000 | 89,374,000 | 21,707,000 |
| M001–4 | 20,225,000 | 45,065,000 | 92,345,000 | 146,472,500 | 251,970,000 | 204,570,000 | 113,237,500 | 40,580,000 | 5,062,500 |
| M002 | 1,140,000 | 2,040,000 | 1,920,000 | 1,950,000 | 540,000 | 0 | 0 | 0 | 0 |
| M003 | 1,929,000 | 2,742,000 | 3,987,000 | 4,503,000 | 6,459,000 | 4,506,000 | 2,469,000 | 1,062,000 | 297,000 |
| M004 | 3,120,400 | 7,775,200 | 14,308,000 | 21,973,400 | 31,470,800 | 25,499,000 | 8,384,400 | 0 | 120,880 |
| addition | 30,331,500 | 55,623,830 | 104,590,770 | 179,385,860 | 324,199,220 | 301,126,470 | 175,807,640 | 54,378,270 | 10,821,570 |

(B)

|  | 0–4 years | 5–9 years | 10–14 years | 15–19 years | 20–24 years | 25–29 years | 30–34 years | 35–39 years | 40–44 years | 45–49 years |
| --- | --- | --- | --- | --- | --- | --- | --- | --- | --- | --- |
| M000 | 253,200 | 366,100 | 270,000 | 412,100 | 898,400 | 1,845,200 | 5,282,400 | 12,448,900 | 26,576,700 | 48,275,100 |
| M000–2 | 0 | 0 | 33,360 | 246,030 | 871,530 | 946,590 | 1,010,530 | 1,170,450 | 1,549,850 | 2,147,090 |
| M001 | 1,995,600 | 2,995,380 | 3,246,240 | 3,325,200 | 4,858,680 | 13,002,720 | 38,839,140 | 91,255,080 | 198,307,980 | 357,413,940 |
| M001–2 | 0 | 0 | 1,000,000 | 600,000 | 1,700,000 | 1,500,000 | 3,250,000 | 3,750,000 | 8,100,000 | 14,150,000 |
| M001–3 | 0 | 0 | 0 | 0 | 1,125,000 | 1,854,000 | 3,161,000 | 8,081,000 | 12,832,000 | 27,541,000 |
| M001–4 | 2,437,500 | 3,937,500 | 2,250,000 | 1,875,000 | 0 | 0 | 0 | 0 | 1,875,000 | 7,500,000 |
| M002 | 360,000 | 300,000 | 0 | 0 | 510,000 | 480,000 | 510,000 | 630,000 | 870,000 | 1,230,000 |
| M003 | 0 | 0 | 0 | 0 | 0 | 0 | 297,000 | 801,000 | 1,635,000 | 3,021,000 |
| M004 | 0 | 0 | 0 | 0 | 0 | 0 | 2,612,000 | 5,394,000 | 9,149,000 | 14,498,000 |
| addition | 875,940 | 1,499,750 | 1,055,270 | 993,550 | 750,930 | 2,307,980 | 6,977,110 | 16,669,050 | 40,324,830 | 76,313,650 |

|  | 50–54 years | 55–59 years | 60–64 years | 65–69 years | 70–74 years | 75–79 years | 80–84 years | 85–89 years | ≥90 years |
| --- | --- | --- | --- | --- | --- | --- | --- | --- | --- |
| M000 | 52,726,500 | 47,874,000 | 49,033,500 | 51,593,300 | 66,573,600 | 48,796,600 | 33,119,600 | 17,813,900 | 6,661,700 |
| M000–2 | 2,348,230 | 1,893,700 | 1,491,720 | 1,559,760 | 1,915,840 | 1,265,180 | 529,610 | 180,700 | 0 |
| M001 | 388,064,880 | 350,650,680 | 362,071,620 | 377,750,940 | 469,310,820 | 337,722,840 | 224,620,800 | 114,628,320 | 40,228,800 |
| M001–2 | 20,600,000 | 22,050,000 | 23,600,000 | 28,000,000 | 39,950,000 | 31,750,000 | 19,600,000 | 9,000,000 | 2,500,000 |
| M001–3 | 36,405,000 | 39,401,000 | 51,004,000 | 62,091,000 | 94,334,000 | 93,743,000 | 86,656,000 | 57,523,000 | 17,465,000 |
| M001–4 | 9,937,500 | 9,562,500 | 12,937,500 | 20,812,500 | 35,625,000 | 30,187,500 | 30,375,000 | 17,250,000 | 2,625,000 |
| M002 | 1,140,000 | 1,350,000 | 1,410,000 | 1,560,000 | 480,000 | 0 | 0 | 0 | 0 |
| M003 | 4,350,000 | 4,674,000 | 4,323,000 | 4,485,000 | 4,734,000 | 3,450,000 | 1,980,000 | 1,128,000 | 315,000 |
| M004 | 17,784,000 | 18,809,000 | 17,146,000 | 16,002,000 | 22,088,000 | 18,227,000 | 13,718,000 | 5,623,000 | 936,000 |
| addition | 84,520,520 | 74,588,870 | 75,902,870 | 78,264,460 | 97,606,430 | 70,264,900 | 47,146,740 | 23,961,290 | 7,586,670 |

These data correspond to the graph in Figure 6.
